# CD100 Up-Regulation Induced by Interferon-α on B Cells Is Related to Hepatitis C Virus Infection

**DOI:** 10.1371/journal.pone.0113338

**Published:** 2014-12-01

**Authors:** Yu He, Yonghong Guo, Yun Zhou, Ying Zhang, Chao Fan, Guangxi Ji, Yu Wang, Zhiyuan Ma, Jianqi Lian, Chunqiu Hao, Zhi Q. Yao, Zhansheng Jia

**Affiliations:** 1 Department of Infectious Diseases and Center of liver Diseases, Tangdu Hospital, the Fourth Military Medical University, Xi′an, Shaanxi, People's Republic of China; 2 Department of Internal Medicine, Division of Infectious Diseases, James H. Quillen College of Medicine, East Tennessee State University, Johnson City, Tennessee, United States of America; Temple University School of Medicine, United States of America

## Abstract

**Objectives:**

CD100, also known as Sema4D, is a member of the semaphorin family and has important regulatory functions that promote immune cell activation and responses. The role of CD100 expression on B cells in immune regulation during chronic hepatitis C virus (HCV) infection remains unclear.

**Materials and Methods:**

We longitudinally investigated the altered expression of CD100, its receptor CD72, and other activation markers CD69 and CD86 on B cells in 20 chronic HCV-infected patients before and after treatment with pegylated interferon-alpha (Peg-IFN-α) and ribavirin (RBV) by flow cytometry.

**Results:**

The frequency of CD5^+^ B cells as well as the expression levels of CD100, CD69 and CD86 was significantly increased in chronic HCV patients and returned to normal in patients with sustained virological response after discontinuation of IFN-α/RBV therapy. Upon IFN-α treatment, CD100 expression on B cells and the two subsets was further up-regulated in patients who achieved early virological response, and this was confirmed by *in vitro* experiments. Moreover, the increased CD100 expression via IFN-α was inversely correlated with the decline of the HCV-RNA titer during early-phase treatment.

**Conclusions:**

Peripheral B cells show an activated phenotype during chronic HCV infection. Moreover, IFN-α therapy facilitates the reversion of disrupted B cell homeostasis, and up-regulated expression of CD100 may be indirectly related to HCV clearance.

## Introduction

Hepatitis C virus (HCV) infection is a major public health problem. The persistence of virus infection increases the risk of end-stage liver diseases, such as liver cirrhosis and hepatocellular carcinoma [1]. Before administration of direct-acting antiviral agents, the standard therapy for chronic hepatitis C has been based on pegylated interferon-α (Peg-IFN-α) and ribavirin (RBV), which provides sustained inhibition of the infection in 40%–55% of patients [2]. According to China's economy, Peg-IFN-α and RBV are mainly anti-HCV agents in recent years. Therefore, it is important to understand the mechanisms of IFN-α-based anti-HCV therapy. In addition to direct inhibition of viral replication [3], IFN-α likely exerts immunomodulatory activities on the elimination of HCV-infected cells [4], [5]. Abundant studies have explored the mechanisms of T cells, NK cells and monocyte-function alterations in the course of antiviral treatment [4], [6]–[10], whereas the mechanisms underlying IFN-α-mediated B-cell immunity during chronic HCV infection remains to be further elucidated.

Semaphorin family members are traditionally involved in neuronal development and axonal guidance. In 1996, CD100, also called Sema4D, was the first semaphorin protein found to have immunoregulatory functions [11], [12]. In the immune system, CD100 is constitutively expressed on resting T cells and natural killer (NK) cells and weakly expressed on B cells and dendritic cells, which promotes immune cell activation and responses [12]–[23]. These processes are primarily mediated via interactions between CD100 and its receptor, CD72 [12]–[15], [24]. Binding of CD100 to CD72 enhances immune responses by reversing the negative signaling effects of CD72 [13], [24]. Several lines of evidence show that CD100 plays an important role in the humoral and cellular immune responses [14], [16], [23]. Recently, it has been reported that CD100 is involved in immune cell responses during human immunodeficiency virus (HIV) and hantaan virus (HTNV) infection [25], [26], indicating that viral infection might also affect CD100 expression and its related immune responses. However, the knowledge of functional roles of CD100 in infectious disease is very restricted. Related studies focused on CD100 and HCV infection have been not reported so far.

In this study, we employed 20 chronic HCV-infected patients before and after antiviral treatment to determine the roles of HCV and IFN-α on CD100 and CD72 expression in B cells. We found that HCV infection and IFN-α therapy could up-regulate CD100 expression, which declined to the normal level in HCV patients who achieved sustained virological response (SVR). Importantly, IFN-α-induced CD100 expression on B cells was negatively correlated with the HCV RNA level, suggesting that enhanced CD100 expression might be associated with the control of HCV infection.

## Materials and Methods

### Study cohort

Peripheral B lymphocytes were studied in 20 patients with chronic HCV infection (anti-HCV+/HCV-RAN+) and 17 age- and sex-matched healthy controls. Twenty HCV patients were treated with Peg-IFN-α-2a (Pegasys, Roche) and RBV for 6–12 months, depending on the different genotypes, and all of them achieved an early virological response (EVR, defined as serum HCV RNA being undetectable, <100 copies/ml, at week 12) and sustained virological response (SVR, defined as HCV RNA remaining undetectable after discontinuation of treatment for at least 6 months), respectively. Basic information on the HCV patients and healthy subjects are described in Table 1. All treatment-naïve patients tested positive for anti-HCV by enzyme-linked immunosorbent assay (Kechuang and Xinhua, Shanghai, China). HCV RNA titers were measured using a fluorescent quantitative transcription polymerase chain reaction (FQ-PCR) assay (Qiagen, Shenzhen, China), with a lower limit of detection of 100 copies/mL. Patients co-infected with hepatitis B, hepatitis D and HIV were excluded. These studies were approved by the Research and Ethical Committee of Tangdu Hospital of the Fourth Military Medical University, and all subjects gave written informed consent in accordance with the Declaration of Helsinki.

**Table 1:** Clinical characteristics of the study population.

	HC	HCV	EVR	SVR
	(n = 17)	(n = 20)	(n = 20)	(n = 20)
Age, year[mean±SEM]	35.82±2.89	45.4±2.97	45.4±2.97	45.4±2.97
Sex, Female/male	9/8	10/10	10/10	1010
ALT, IU/ml[mean±SEM]	n.a.	85.9±18.76	32.35±5.41	23.3±2.16
HCV-RNA mean log10cps/ml±SEM	n.a.	5.97±0.15	<2.0	<2.0
HCV genotype 1b/2a	n.a.	10/7	10/7	10/7

Abbreviations: HC: healthy controls; HCV: treatment-naive patients with chronic hepatitis C; EVR: HCV patients with EVR after 3-month antiviral treatment; SVR: patients who achieved SVR after antiviral treatment; SEM: standard error of mean; n.a.: not applicable. Note: HCV genotype could not be identified in 3 patients in HCV, EVR and SVR group.

### Flow cytometry

PBMCs were isolated by Ficoll-Hypaque density centrifugation (Sigma, St Louis, MO) according to the manufacturer's protocol. PBMCs were stained with anti-CD19-PeCy5.5, anti-CD5-PE, anti-CD72-FITC, anti-CD69-FITC (BD Biosciences, San Jose, CA), anti-CD86-PE (Biolegend, San Diego, CA) and anti-CD100-APC (R&D Systems) or isotype-matched control (BD Biosciences) monoclonal antibodies. Stained cells were analyzed on a multi-color Arial II with FACS Diva version 6.1.3 (BD Biosciences) and the FlowJo Version 7.6 software.

### Preparation of HCV

HCV FL-J6/JFH (kindly provided by Dr. C. Rice, The Rockefeller University, New York, NY) was produced as described [27]. The plasmid DNA was linearized with XbaΙ (Fermentas, Vilnius, LTU). Purified and linearized DNA was transcribed into mRNA using a TranscriptAid T7 High Yield Transcription kit (Fermentas, Vilnius, LTU), and the transcribed mRNA was transfected into Huh7.5 cells (provided by Dr. C. Rice) using the DMRIE-C reagent (Invitrogen, Carlsbad, CA) per the company's instructions. The 48-h supernatant of FL-J6/JFH RNA-transfected Huh7.5 cells was collected to infect naïve Huh7.5 cells to produce HCV particles. The HCV RNA titer was determined as previously described [28].

### HCV infection

PBMCs from healthy controls were incubated with HCV particles or UV-inactivated HCV virions (UV-HCV) at a multiplicity of infection (MOI) of 10 or with HCV core protein (ViroGen, Watertown MA) at a concentration of 2 µg/ml for 48 h. Complete cell medium or human IgG was used as the negative control. After incubation, the cells were stained with anti-CD19, CD5, CD100 and CD72 monoclonal antibodies for flow cytometric analysis.

### Treatment of PBMCs with IFN-α

The expression of CD100 was assessed in B cells that were or were not stimulated with recombinant IFN-α-2a (Roche, Switzerland). Frozen PBMCs were thawed and washed twice with RPMI 1640. PBMCs (0.5×106 cells) were used directly for cell surface staining or incubated in 1 ml complete medium supplemented with IFN-α-2a (at a concentration ranging from 0.01 to 1000 ng/ml) in a 24-well, round-bottom plate (Corning, NY) for 2, 6, 12, 24 and 48 h. Complete cell medium was used as the negative control. After incubation, PBMCs were stained with anti- CD19, CD5, CD100 and CD72 and analyzed by flow cytometry.

### Statistical analysis

Statistical analyses were performed using GraphPad Prism Version 5.0 (GraphPad). Mann-Whitney U tests were employed to compare treatment-naïve patients with healthy controls, and the Paired t-test or Wilcoxson matched-pairs test were used for paired variables, depending on the data distribution. Relationships between CD100 and clinical parameters were evaluated using the Spearman rank correlation test. *P*<0.05 was considered significant for all tests.

## Results

### High frequency of the peripheral B cell-activated phenotype in chronic HCV patients

**Figure 1:**
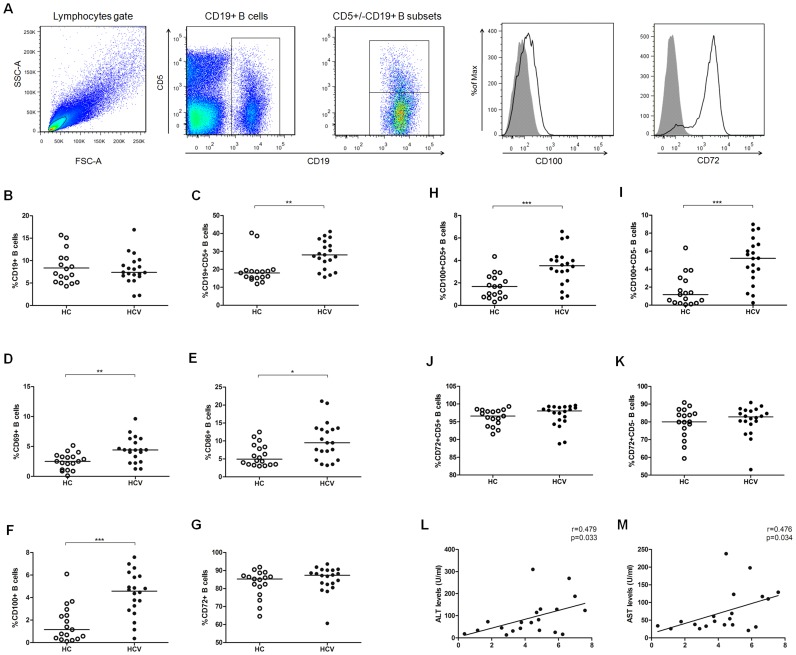
Disrupted B cell homeostasis in patients with chronic HCV infection. (A) Gating strategy. (B) The frequencies of CD19+ B and (C) CD5+CD19+ B cells in 20 HCV-untreated patients (HCV, filled circles) and 20 healthy controls (HC, open circles). (D) The CD69, (E) CD86, (F) CD100 and (G) CD72 expression levels on B cells in HCV patients and healthy controls. The distribution of (H–I) CD100 and (J–K) CD72 in CD5+ and CD5- B cell subsets. The expression of CD100 on B cells was positively correlated with (L) ALT or (M) AST levels.

To determine the effects of HCV infection on B cells, we assessed the frequency of B cells and their subsets in HCV patients using flow cytometric analysis. Fig.1A shows the gating strategy as dot plots and CD100/CD72 expression as histograms of a representative experiment. The frequency of CD19^+^ B cells was equal in HCV patients and healthy controls (Fig. 1B), whereas the percentage of CD5^+^CD19^+^ B cells in HCV patients was significantly higher than in healthy controls (Fig. 1C). Next, we evaluated the expression of activation markers on B cells, including the early activation molecule CD69 and the co-stimulatory molecules CD86 and CD100. The percentages of these molecules on B cells were significantly increased in patients with chronic HCV infection compared with healthy controls (Fig. 1D–F). We also examined the expression of the CD100 receptor, CD72, on B cells (a negative regulator), but no significant differences were observed between HCV patients and healthy controls (Fig. 1G). CD100 showed a higher frequency of distribution in CD5^−^ B cells, whereas CD72 expression was mainly on the CD5^+^ B subsets (Fig. 1H–K). To identify whether CD100 was involved in viral control and disease progression, we analyzed the relationships between CD100 and clinical parameters, such as alanine aminotransferase (ALT), aspartate aminotransferase (AST) and HCV-RNA, and found that CD100 expression was positively related to serum ALT (r = 0.479, p = 0.033) and AST levels (r = 0.476, p = 0.034) (Fig. 1L and 1M), but not to HCV-RNA (data not shown). These data suggested that peripheral B cells in chronic HCV patients show an activated phenotype and are associated with hepatic immune-mediated inflammation.

### IFN-α-based therapy reversed disrupted B cell homeostasis

**Figure 2:**
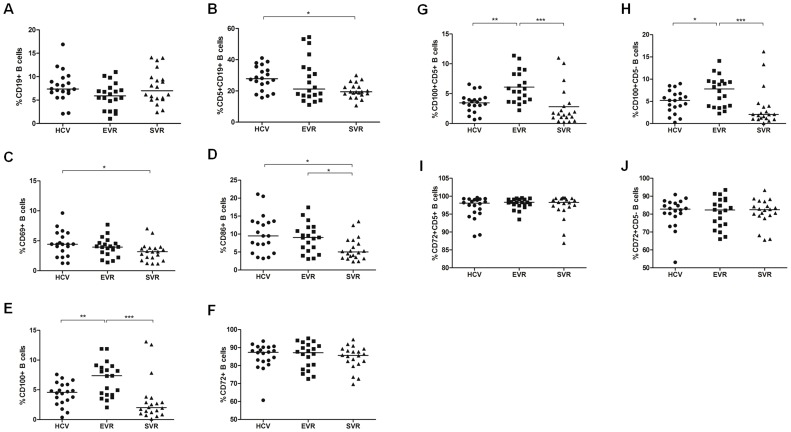
The percentages and phenotypes of altered B cells and their subsets in HCV patients after initiation of IFN-α/RBV treatment. The frequency of (A) CD19+ B and (B) CD5+CD19+ B cells in patients with chronic HCV and healthy controls. The changes in (C) CD69, (D) CD86, (E) CD100 and (F) CD72 expression on B cells in chronic HCV-infected patients before and after antiviral treatment. (G–H) The expression of CD100 and (I–J) CD72 on the CD5+ and CD5- B cell subpopulations after initiation of IFN-α therapy. HCV (filled circles), EVR (filled squares) and SVR (filled triangles).

To determine the roles of IFN-α treatment on the altered B-cell expansion and activation, we performed a longitudinal analysis to investigate the percentages and phenotypes of B cells and their subsets in HCV-infected patients before and after antiviral treatment. There were no significant changes in the frequency of CD19^+^ B cells among the three groups (HCV, EVR and SVR), whereas the proportion of CD5^+^CD19^+^ B cells as well as CD69 and CD86 expression slightly decreased in EVR patients and further declined to normal levels in SVR patients (Fig. 2A–D). Interestingly, in contrast to CD69 and CD86, CD100 expression on B cells and both subsets was further up-regulated in EVR patients, and the levels returned to normal in patients with SVR following IFN-α treatment (Fig. 2E, G and H). In contrast, the expression of CD72 on both B subpopulations did not markedly change upon IFN-α therapy (Fig. 2F, I and J).

### CD100 expression on B cells might be associated with the virological response during early-phase IFN-α therapy

**Figure 3:**
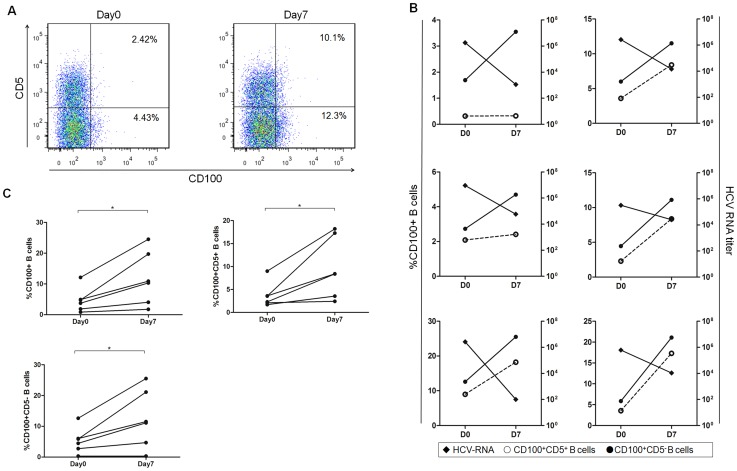
Correlation analysis between CD100 expression on B cells and serum HCV-RNA levels during the early phase of IFN-α treatment. (A) Representative dot plots of the CD100 percentages in CD5+ and CD5- B cell subsets in one of the six HCV patients are shown. (B) The CD100 expression on CD5+ (open circles) and CD5- B cells (filled circles) and serum HCV-RNA levels (filled rhombs) immediately before and seven days after IFN-α/RBV treatment is shown for six chronic HCV-infected patients.

To identify whether CD100 was related to the virological response, we examined the relationship between CD100 expression and the HCV RNA level in HCV patients undergoing early-phase IFN-α/RBV therapy. Six blood samples were collected on days 0 and 7 of treatment, and the HCV RNA titer in the serum was quantified. As expected, HCV viral titers were suppressed, whereas CD100 expression on B cells and their subsets was significantly increased after antiviral treatment (Fig. 3A–C). More importantly, the up-regulation of CD100 expression on B cells inversely correlated with the HCV-RNA decline following antiviral treatment (Fig. 3B). Thus, CD100 expression on B cells seemed to correlate with the virological response. This led us to address whether *in vitro* IFN-α treatment could up-regulate CD100 expression on B cells.

### The effects of HCV and IFN-α on CD100 expression by B cells *in vitro*

**Figure 4:**
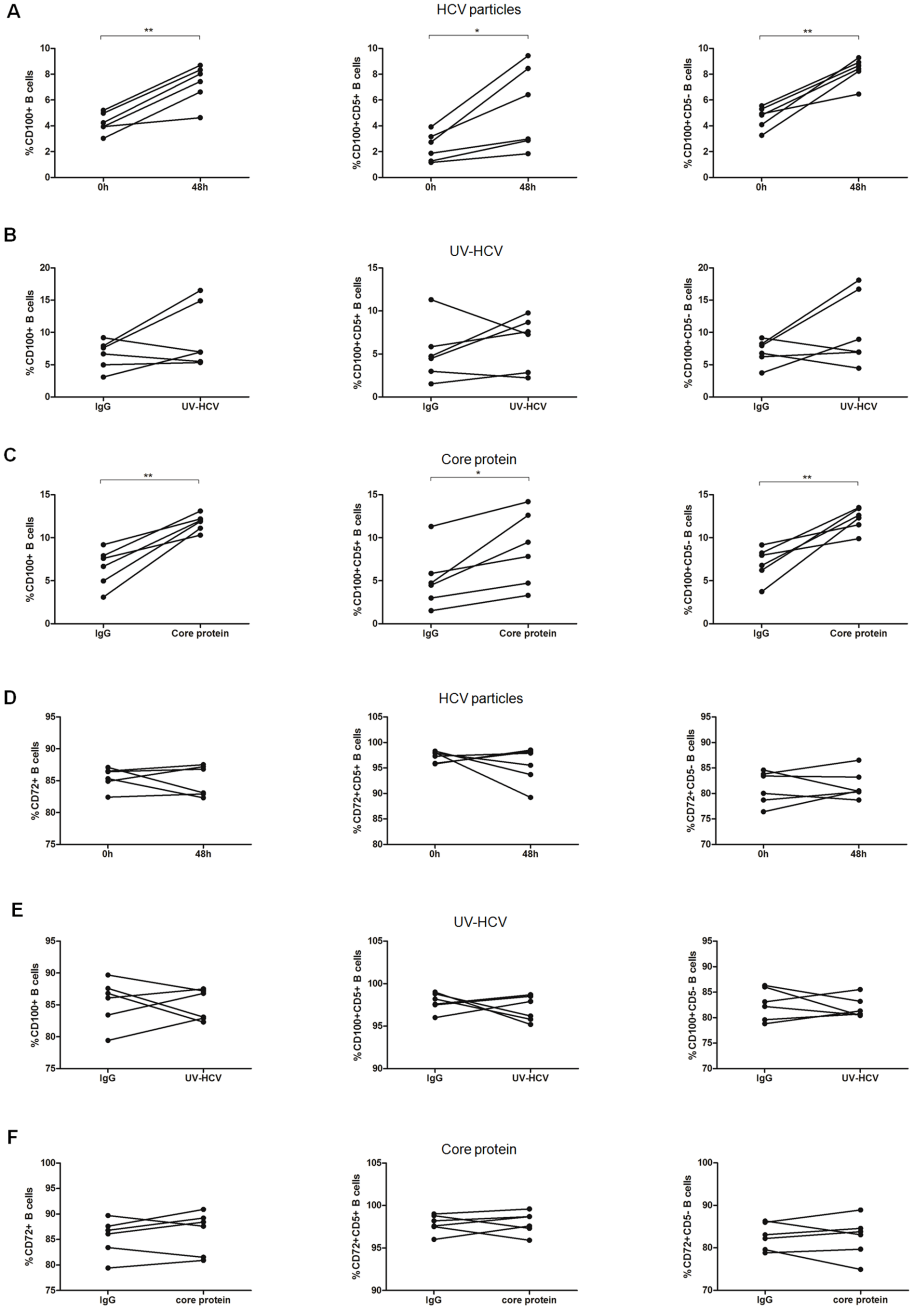
HCV could up-regulate CD100 expression on B cells and their subsets. (A–C) CD100 and (D–F) CD72 expression on total, CD5+ and CD5- B cells after stimulation of PBMCs with HCV particles, UV-HCV or HCV core protein for 48 h.

**Figure 5:**
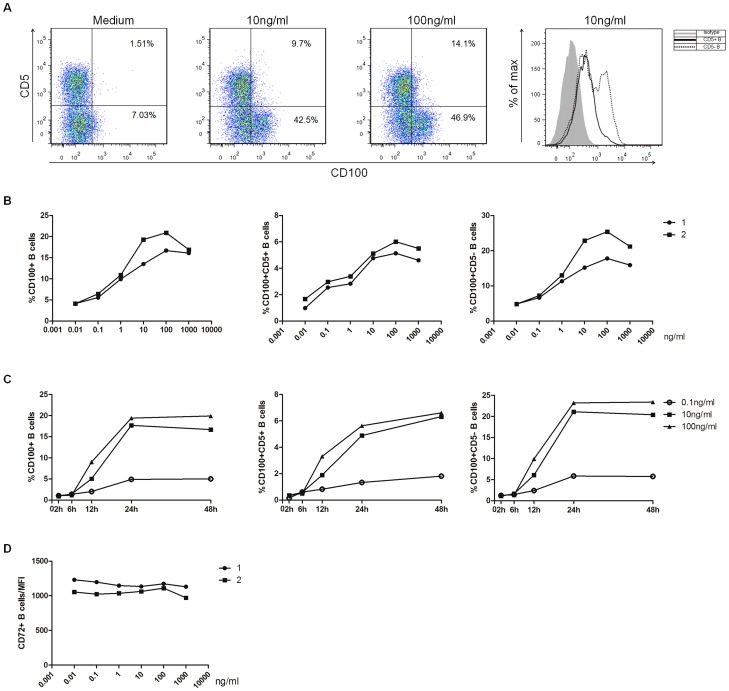
The effect of IFN-α on CD100 expression in B cells and their subpopulations. (A) CD100 expression on CD5+ and CD5- B cells from one representative, healthy donor before and after IFN-α stimulation of PBMCs. (B) CD100 expression alterations in B cells and their subsets after 24-h stimulation with 0.01, 0.1, 1, 10, 100 or 1000 ng/ml of IFN-α. Two of six representative individuals are shown. (C) CD100 expression at different time points: 0, 2, 6, 12, 24 and 48 h on B cells and their subsets after stimulation with IFN-α at a concentration of 0.1 ng/ml (open circles), 10 ng/ml (filled squares) or 100 ng/ml (filled triangles). One of the six representative individuals is shown. (D) The mean fluorescence intensity (MFI) of CD72 expression on B cells stimulated with different concentrations of IFN-α. Two of six representative individuals are shown.

To identify the etiology of the increased CD100 expression in patients with HCV infection, we carried out *in vitro* studies to confirm whether HCV particles, UV-HCV or the HCV core protein affected CD100 and CD72 expression on B cells. As expected, CD100 expression on total B cells and the two subsets, particularly CD5^-^B cells, was significantly increased after 48-h stimulation of PBMCs with HCV particles or core protein compared to human IgG (Fig. 4A and 4B), which was consistent with the *ex vivo* results (Fig. 1F, H and I), but not with UV-HCV (Fig. 4C). Next, we examined the impact of IFN-α on CD100 expression. Similarly, CD100 expression on B cells and their subsets was notably up-regulated in a dose-dependent manner after 24-h stimulation of PBMCs with IFN-α (Fig. 5A and B). Indeed, CD100 expression began to rise at 6 h and peaked at 24 h following IFN-α treatment (Fig. 5C). IFN-α seemed to have a stronger effect on CD100 expression in CD5^-^ B cells compared to in CD5^+^ B cells. However, CD72 expression on B cells was not affected by HCV particles, UV-HCV, the core protein (Fig. 4D–F) or IFN-α (Fig. 5D). These data suggested that up-regulation of CD100 was mainly manipulated by the virus during chronic HCV infection, whereas this increase was primarily achieved by IFN-α rather than HCV after the initiation of antiviral treatment.

## Discussion

Several studies have reported that CD100 has immunomodulatory activities in the humoral and cellular immune responses [14], [16]–[18], [22], [23], [29], which is significantly up-regulated after cellular activation [11]–[13]. Recently, Eriksson EM et al have demonstrated that CD100 is also involved in the T-cell response during HIV infection [25]. However, it is still unclear whether chronic HCV infection affects CD100 expression and the related immune response. Here, we assessed the frequency and phenotype of B cells in HCV patients treated with IFN-α and found HCV infection and IFN-α up-regulated CD100 expression. Importantly, increased CD100 expression was inversely correlated with HCV RNA decline. Our results suggest that CD100 expression on B cells might be associated with the control of HCV infection.

To the best of our knowledge, no related studies have focused on the relationship between CD100 and HCV infection. Several lines of evidence have shown that HCV infection is usually associated with disturbances of B lymphocyte activation and polyclonal proliferation [30]–[34]. In line with several studies [33], [35]–[37], the percentage of CD5^+^CD19^+^ B cells was increased in HCV patients compared with healthy controls, and, consistent with other studies [30], [38], we showed that circulating B lymphocytes in HCV patients presented an activated phenotype with increased CD69, CD86 and CD100 expression. In addition, our *in vitro* results showing that HCV particles and core protein could up-regulate CD100 expression, but not UV-HCV. It has been reported that HCV replication exists in B lymphocytes and sustained HCV-driven antigenic stimulation may play a vital role in B cell proliferation and activation [30], [39]–[41]. Therefore, we speculated that HCV particles entry into B cells via entry receptors and replicate and then regulate CD100 expression. It is known that B cells express gC1qR and CD81, which are the receptors of HCV core protein and E2 protein, respectively [42]. Then, UV-HCV, as compound protein (E1 and E2 protein), and core protein interacting with their receptors might regulate CD100 expression through different mechanisms. However, the underlying mechanisms need to be further elucidated through more researches. As is known, CD5, which is expressed on a subset of B cells, is associated with the production of low-affinity immunoglobulin M and regulation of the immune response [43]. Therefore, our results suggest that expansion of CD100^+^ and CD5^+^ B cells might play a role in pathogenesis due to viral-specific activation and proliferation during chronic HCV infection.

**Figure 6:**
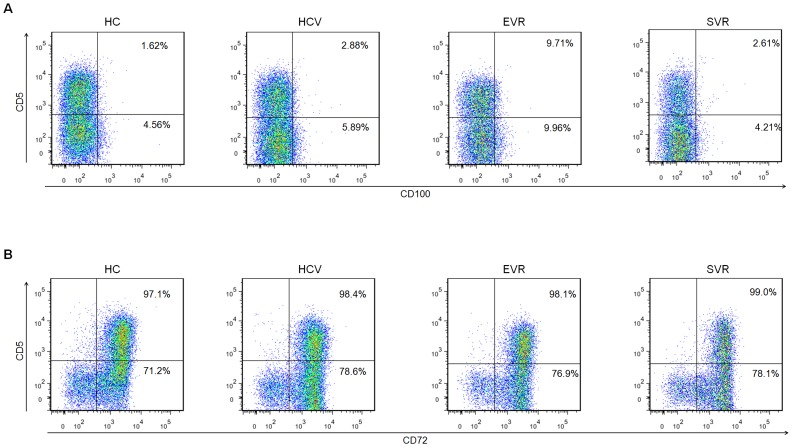
Representative dot plots of CD100 and CD72 expression in HCV patients and healthy controls. (A) CD100- and (B) CD72-expressing CD5+ and CD5- B cell subpopulations in healthy controls (HC), chronic HCV patients and patients with EVR and SVR.

Next, we investigated the alteration of the B-cell frequency and phenotype during antiviral treatment. The percentage of CD5^+^CD19^+^ B cells as well as the expression of the activation markers CD69 and CD86 did not significantly change in EVR patients, but returned to normal levels in SVR patients following IFN-α treatment. One explanation for this phenomenon might be that residual HCV RNA might exist in B cells even though the viral RNA was undetectable in the plasma. Thus, persistent stimulation of residual virus still acted on B cells until the disrupted B cell homeostasis recovered concurrent with complete HCV-RNA clearance in SVR patients. Interestingly, in contrast to CD69 and CD86, CD100 expression was further up-regulated following 7-day and 3-month IFN-α/RVB therapy, which was confirmed by *in vitro* experiments using PBMCs stimulated with IFN-α. To mimic the complex *in vivo* conditions during therapy, we employed PBMCs rather than isolated B cells to examine CD100 expression upon stimulation by IFN-α *in vitro*. Therefore, we can′t rule out whether other immune cells are involved in the regulation of CD100 expression. After all, B cells need to interact with other cells to induce immune response to HCV infection *in vivo*. Through these results, we found that increased CD100 expression on B cells in EVR patients was primarily regulated by IFN-α, rather than HCV. According to the changing trend of CD100 expression in HCV patients before and after antiviral treatment, we propose that CD100 induced by IFN-α may play a functional role in the B cell immunoregulation in response to HCV infection (Fig. 6).

Through correlation analysis, we showed that the CD100 expression on B cells was positively related to the ALT and AST levels in HCV treatment-naïve patients, whereas the expression of CD100 was negatively related to the HCV RNA titer during early phases of antiviral treatment. These results suggest that CD100 expression on B cells seemed to have a functional role in immune-mediated inflammation and the virological response. In addition to regulating humoral responses, B cells are also important antigen-presenting cells. We have proven that the CD100 receptor, CD72, is expressed on CD8^+^ T cells (data not shown); therefore, as a co-stimulatory molecule, increased B cell-CD100 ligation with T cell-CD72 might facilitate CD8^+^ T cell responses by turning off CD72-negative signal effects during antiviral treatment. Our further research will focus on identifying how T cells exert their killing function via CD100.

Previous studies have revealed that soluble CD100 retained the same biological activities as membrane-bound CD100 [44]. Hence, one question arising from this study was whether sCD100 could be used therapeutically in combination with other antiviral agents to treat chronic HCV-infected patients. Further studies are needed before sCD100 is applied clinically.

One limitation of our study was that non-responders to IFN-α were not included in our study, and this group would have been ideal to better dissect the effect of IFN-α on B cells and HCV clearance. Another caveat was that we lacked sufficient evidence that supports a direct role of CD100 on B cells in the control HCV infection. Therefore, additional studies are necessary to further solve these issues.

In conclusion, we evaluated the expression pattern of CD100 and its receptor, CD72, on B cells during chronic HCV infection. We showed a novel mode of action, whereby IFN-α therapy enhanced the expression of CD100 on B cells, and this might be associated with HCV clearance.
